# Electroacupuncture as an eosinophil-targeting treatment in ovalbumin-induced allergic rhinitis involving β_2_-adrenergic receptor in a mouse model

**DOI:** 10.3389/fimmu.2026.1832773

**Published:** 2026-06-16

**Authors:** Tran Van Bao Quach, Thanh-Hien Vu Nguyen, Ngoc Chi Lan Nguyen, Che-Hsuan Lin, Yi-Hung Chen

**Affiliations:** 1 Graduate Institute of Acupuncture Science, China Medical University, Taichung, Taiwan; 2 Faculty of Traditional Medicine, Can Tho University of Medicine and Pharmacy, Can Tho, Vietnam; 3 Graduate Institute of Dental Sciences, China Medical University, Taichung, Taiwan; 4 Department of Otolaryngology, School of Medicine, College of Medicine, Taipei Medical University (TMU), Taipei, Taiwan; 5 Department of Otolaryngology, Taipei Medical University Hospital, Taipei Medical University, Taipei, Taiwan; 6 International Master Program in Integrative Health, China Medical University, Taichung, Taiwan; 7 Chinese Medicine Research Center, China Medical University, Taichung, Taiwan, Taiwan; 8 Department of Computer Science and Information Engineering, Asia University, Taichung, Taiwan

**Keywords:** allergic rhinitis, electroacupuncture, eosinophils, epinephrine, norepinephrine, β2 adrenergic receptors

## Abstract

**Background:**

Eosinophils amplify type-2 (Th2) inflammation and tissue injury in allergic rhinitis (AR), and eosinophilic burden correlates with disease severity and future asthma risk. Current AR therapies have limitations, motivating interest in non-pharmacologic neuromodulatory approaches. Here, we tested whether electroacupuncture (EA) attenuates eosinophilic inflammation in AR and probed a candidate neuroimmune mechanism.

**Methods:**

Using an ovalbumin (OVA)-induced AR mouse model, we compared EA with the antihistamine chlorpheniramine (CLP). We assessed nasal behaviors, inflammatory biomarkers, and histological changes. Mechanistic exploration involved administering β_2_-adrenergic (butoxamine) or dopamine D1 (butaclamol) antagonists before EA, followed by plasma catecholamine measurements and intranasal epinephrine rescue.

**Results:**

In OVA-challenged mice, EA significantly alleviated nasal rubbing, redness, and olfactory dysfunction, showing comparable efficacy to CLP. While OVA induction increased IL-5, IL-13, and serum OVA-specific IgE, both treatments significantly reduced these markers. Crucially, only EA reversed OVA-induced nasal eosinophil infiltration and suppressed RNASE2A expression; CLP primarily suppressed mast cell degranulation and MCPT1 expression. Mechanistically, pre-treatment with butoxamine—but not butaclamol—abolished the EA-mediated reduction of OVA-induced IL-5, IL-13, RNASE2A, and CCR4. Furthermore, EA was associated with elevated plasma norepinephrine and epinephrine levels. While butoxamine blocked EA-induced symptom relief and eosinophil reduction, intranasal epinephrine mimicked EA’s beneficial effects on these parameters.

**Conclusions:**

Our findings demonstrate that EA reduces eosinophilic inflammation and AR behaviors associated with the activation of β_2_-adrenergic receptors. Unlike standard antihistamines that target mast cells, EA engages a sympathetic neuroimmune axis, representing a promising complementary intervention for eosinophil-driven AR.

## 1 Introduction

Allergic rhinitis (AR) is T helper cell type 2 (Th2) inflammatory allergic condition, characterized by sneezing, nasal congestion, rhinorrhea, and itching. AR affects 10–40% of the global population (1–4), and is the most common Th2 inflammatory disease alongside atopic dermatitis and asthma (2–4). AR develops through allergen sensitization followed by early-phase mast cell activation and late-phase infiltration of Th2 cells, eosinophils, and other inflammatory cells into the nasal mucosa (5, 6). Persistent or poorly controlled AR, particularly with eosinophilic inflammation, has been associated with an increased risk of subsequent asthma (7–11), highlighting the importance of effective inflammatory control.

Among the immune cells involved in AR, eosinophils are key drivers of disease progression and symptom severity. Recruited in part by IL-5, eosinophils amplify Th2 inflammation through cytokine release, antigen presentation to CD4+ T cells, and promotion of Th2 polarization (12, 13). They can also secrete Th2-type cytokines, such as IL-4, and release cytotoxic granule proteins—including eosinophil cationic protein (ECP), eosinophil peroxidase (EPX) (14), and eosinophil-derived neurotoxin (EDN) (13), cause tissue damage, recruit and activate additional Th2 effector cells, thereby sustaining late-phase inflammation (12–14). Clinically, elevated eosinophil activation in blood and nasal tissue is associated with greater symptom severity, particularly in moderate-to-severe AR and in patients with comorbid asthma (14–17). Together, these findings identify eosinophilic inflammation as a central driver of AR and an important therapeutic target.

First-line therapies for AR include H1-antihistamines, intranasal (INS) corticosteroids, and allergen avoidance (5). While these treatments are effective in relieving symptoms, they have notable limitations. Classic antihistamines (e.g., chlorpheniramine) primarily target histamine-mediated pathways and have little effect on eosinophil burden while some second-generation agents (e.g., fexofenadine, cetirizine) can reduce systemic eosinophils (18–21). In contrast, corticosteroids, although effective, may be associated with local irritation and concerns about systemic exposure (22–25). Accordingly, targeting eosinophils has become a recent focus in airway allergic management. Anti–IL-5/anti–IL-5Rα biologics such as mepolizumab and benralizumab reduce eosinophilia and improve outcomes in eosinophilic asthma (26), however they remain costly and are not routinely used for AR (27). These limitations highlight the need for complementary approaches that can modulate type 2 inflammation, particularly eosinophilic responses, in the nasal mucosa.

Acupuncture, with a history spanning over 2,000 years, is widely used for various diseases (28, 29). The remarkably high public engagement and increasing clinical utilization of acupuncture in the ASEAN region and East Asia (30, 31) are now supported by a global rise in high-quality clinical trials (32), regional strategic initiatives for standardization (33), and technological advancements in precision training (34). Acupuncture involves inserting needles into specific sites (acupoints) and stimulating them manually (manual acupuncture) or with electrical current (electroacupuncture) to induce therapeutic effects (35). In AR, randomized and network meta-analyses suggest benefits on symptoms and reduced antihistamine use (36–38), suggesting a potential option for patients who respond poorly to conventional therapy or cannot tolerate side effects (38). Electroacupuncture (EA) which combines needling with low-level electrical stimulation, has also shown a favorable safety profile (39). However, the mechanisms underlying the efficacy of EA in AR remain to be fully understood.

Emerging evidence suggests that EA can engage neuroimmune pathways through autonomic regulation and catecholamine release (40). Catecholamines (epinephrine – EPI, norepinephrine – NE) levels, representing sympathetic–adrenergic tone, appear dysregulated in AR (41, 42). These mediators act through α- and β-adrenergic receptors (α-AdR, β-AdR) to influence vascular responses, adjust T-helper balance, and dampening Th2 cytokine (41, 43–51). Particularly, β_2_-adrenergic signaling reduces ICAM-1 on airway neurons and directly suppresses eosinophil functions *in vitro* (52, 53). These observations raise the possibility that EA may alleviate AR not only by reducing symptoms, but also by directly restraining eosinophilic inflammation through a β_2_-adrenergic mechanism.

In the present study, we used an ovalbumin (OVA)-induced mouse model of AR to investigate whether EA suppresses eosinophilic inflammation and improves AR-like behaviors. We compared the effects of EA with chlorpheniramine (CLP), a first-line antihistamine, and further examined the involvement of β_2_-adrenergic signaling using pharmacologic antagonism and INS EPI administration. We hypothesized that EA would exert a therapeutic profile distinct from antihistamine treatment by selectively reducing eosinophilic inflammation through a β_2_-adrenergic neuroimmune pathway.

## 2 Material and methods

### 2.1 Animals

Female BALB/c mice (4–5 weeks old, 17–19 g, BioLasco Taiwan Co., Ltd.), were housed under standard conditions with free access to food and water. After a 7-day acclimation period, the mice were randomly assigned to experimental groups (n = 6–9). Experimental groups included naive, OVA alone, OVA+EA, OVA+CLP (chlorpheniramine), OVA+BTC+EA (butaclamol), and OVA+BTX+EA (butoxamine), OVA+EPI (Epinephrine). Detailed materials are listed in **Supplementary Table E1**.

### 2.2 OVA induces allergic rhinitis

An intraperitoneal (IP) injection of 200 µg OVA (Sigma-Aldrich) and 2 mg aluminum hydroxide diluted in 100 µL of saline was administered to each mouse (except those in the naive group) three times on days 0, 7, and 14. For the local challenge, a 4 µL solution containing 0.5 mg of OVA diluted in distilled water was delivered to both nares of the mouse nasal cavity via a micropipette after EA or drug administration. INS OVA challenges were administered daily on days 21–27 for the 7-day short-term AR model, or on days 21–49 (5 consecutive days a week in 4 weeks) for the prolonged AR model.

### 2.3 Electroacupuncture treatment

The EA procedure used in this study, including acupoint selection (LI4 and LI11) and stimulation parameters, was adapted from previously published studies (54, 55). For the purposes of this mechanistic animal study, murine LI4 and LI11 were defined using an anatomical transpositional approach based on homologous landmarks corresponding to human Hegu (LI4) and Quchi (LI11) (56), providing reproducible stimulation sites for standardized EA delivery. The murine LI4 is located on the first dorsal interossei, radial to the midpoint of the second metacarpal bone in the forelimb, and LI11 is situated in the depression at the lateral end of the cubital crease in the right forelimb.

EA was performed under 1.5% isoflurane anesthesia using 32-gauge needles inserted 2–3 mm deep at murine LI4 (first dorsal interosseous space) and LI11 (lateral cubital crease) acupoints. Electrical stimulation (2 mA, 2 Hz, 150 μs pulse width) was applied for 20 min using Ito Trio-300 stimulator (Ito, Japan), starting 20 min before INS OVA challenges.

### 2.4 Drug administration

Butaclamol (BTC) (40) and butoxamine (BTX) (57) were selected based on previous studies investigating dopamine- and adrenergic-related mechanisms, and their administration protocols were adapted for the present allergic rhinitis model. Mice were acclimated for 10 min and then received an IP injection of BTC (30 mg/kg) or BTX (10 mg/kg), followed by a 20-min interval before EA. Chlorpheniramine (CLP; 10 mg/kg) (58) was administered by oral gavage 20 min before intranasal OVA challenge. Epinephrine (EPI; 0.005%, 4 μL/mouse) (59) was administered by intranasal instillation 3 min after OVA challenge. All pharmacological agents were sourced from Sigma-Aldrich.

### 2.5 Nasal rubbing behavior observation

After INS challenge with OVA, the mice were placed in a small chamber to observe their nasal rubbing and sneezing behavior for 10 min.

Nasal rubbing occurs when a mouse uses its forelimb or hindlimb to scratch the nose or mystacial vibrissae area. For blinding, the other observers counted the bouts of nasal rubbing behavior (60).

### 2.6 Nose redness evaluation

Within 10 min after the last OVA challenge, each mouse’s nose area was photographed. The color of the nasal tubercles was transformed to an RGB color parameter and scored by two main R and G color categories. Nasal redness, a symptom of allergic irritation, was evaluated on a scale of 1 (pale red) to 4 (dark red) (**Supplementary Figure E1**). The test followed previous studies (61–63).

### 2.7 Olfactory dysfunction test

Following previous studies, 6 hr after the last OVA challenge, the mice were subjected to food restriction for 16–18 hr before the test. The mice were allowed to acclimate for 3 min in a 3 cm bedding layer, after which a teddy cookie was placed at random corners. A longer latency to find cookies indicates olfactory dysfunction (64).

### 2.8 Fresh tissue collection

For baseline and post-treatment catecholamine analysis, submandibular blood was collected into EDTA-coated tubes (Sigma-Aldrich) for anticoagulation within 10 min following a one-time EA session, and plasma was isolated. For all other assays, mice were euthanized under deep isoflurane anesthesia (3.5%) 24 hr after the final INS OVA challenge. Serum samples were collected concurrently for ELISA. The murine nasal mucosa from the septal region was carefully excised using fine forceps and immediately immersed in RNAlater stabilization solution (Thermo Fisher Scientific) for downstream molecular analysis, the excising method was adapted from Dunston’s study (65).

### 2.9 mRNA extraction and quantification via real-time quantitative polymerase chain reaction

Total RNA was extracted from nasal mucosa via the PureLink^®^ RNA Mini Kit (Ambion). Complementary DNA (cDNA) was synthesized from RNA by High-Capacity cDNA Reverse Transcription Kit (Applied Biosystems). qPCR analysis was conducted in a StepOnePlus™ Real-Time PCR System with dye-based SYBR (Fast SYBR™ Green Master Mix, Applied Biosystems). The 2-ΔΔCt method (66) was used to determine the relative gene expression to that of the control group via the use of GAPDH as the reference gene, and the primer sequences for IL-5, IL-13, CCR4, CCR5, MCPT1, RNASE2s, and GAPDH are shown in **Supplementary Table E2**.

### 2.10 Hematoxylin & eosin and toluidine blue staining

The animals were transcardially perfused with PBS at the time of sacrifice, followed by 4% paraformaldehyde in PBS. After overnight postfixation at 4 °C in 4% paraformaldehyde, the heads were trimmed, decalcified, embedded in paraffin, and coronally sectioned throughout the entire anterior-posterior extent of the nasal cavity. The sections were then processed and stained with H&E or toluidine blue for eosinophils and mast cells. The staining protocol followed the reference protocol, and the location of the transverse section was determined at the level of the incisive papilla and the duct connecting the nasal and oral cavities (67). The stained sections were scanned via a NanoZoomer-XR digital slide scanner and processed via its viewing platform (NDP.view2).

### 2.11 Enzyme-linked immunosorbent assay

The plasma NE and EPI concentrations were measured by Catecholamine high-sensitivity ELISA kit(R&D) (40). The OVA-IgE kit (Cayman) was used to measure the specific OVA allergen IgE in the serum.

### 2.12 Statistical analysis

Data are presented as mean ± standard error of the mean (SEM). Group comparisons were performed using one-way ANOVA with Holm-Šídák’s *post hoc* test, comparing OVA+EA against OVA and antagonist-treated groups. Redness scores were analyzed using the Kruskal-Wallis test with Dunn’s *post hoc* test. Two-way ANOVA was used for catecholamine changes. Significance was set at p < 0.05, and analyses were conducted in GraphPad Prism 9.0.

## 3 Results

### 3.1 EA at LI4+LI11 mitigates OVA-induced AR symptoms, comparable to CLP

**Figure 1:**
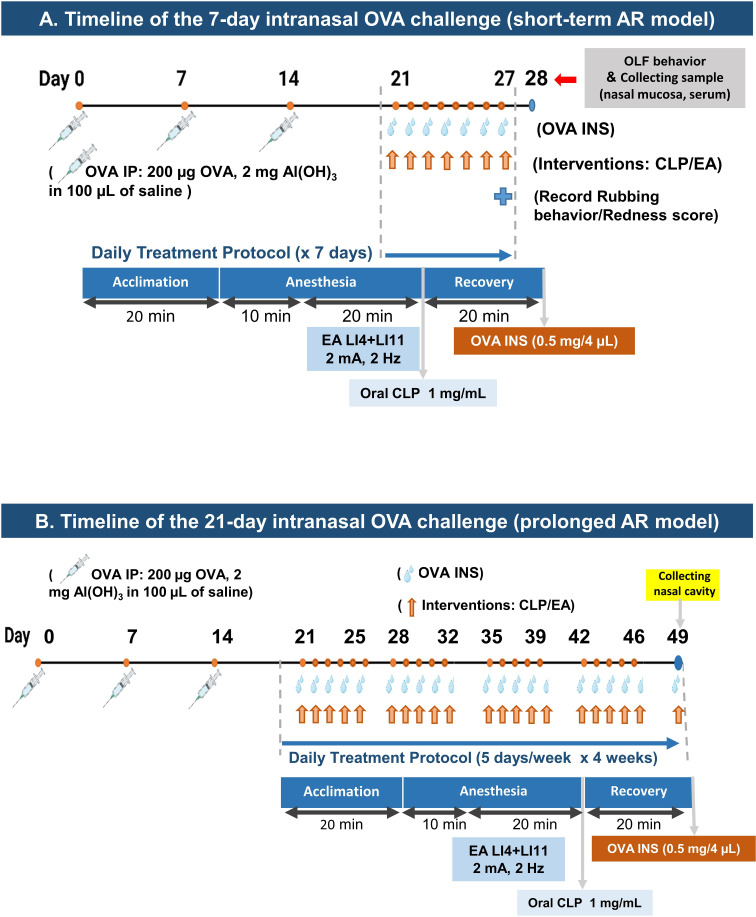
Experimental timelines for the short-term and prolonged ovalbumin (OVA)-induced allergic rhinitis (AR) mouse models. BALB/c mice underwent intraperitoneal (IP) sensitization with OVA and aluminum hydroxide (Al(OH)_3_) on days 0, 7, and 14. **(A)** In the short-term AR model: mice received daily intranasal (INS) OVA challenges for 7 consecutive days (Days 21–27, total 21-day challenge). Daily treatments—either oral chlorpheniramine (CLP) or electroacupuncture (EA)—were administered 20 min prior to the OVA challenge. Behavioral testing and sample collection occurred on Day 28. **(B)** The prolonged AR model: mice received daily INS OVA challenges for 4 weeks (Days 21–49, total 21-day challenge). Treatments (CLP or EA) were administered 5 days per week during this 4-week challenge phase. Nasal cavities were collected on Day 49 for histological analysis.

To mimic AR symptoms and their impact on nasal and olfactory function, we employed three behavioral tests: nasal rubbing to reflect itching, nose redness as a marker of visible inflammation and irritation (61, 62), and the buried food pellet test to evaluate olfactory dysfunction (68, 69). Mouse AR models are generally established by systemic sensitization with repeated IP injections of OVA, followed by local INS challenges (70). Short INS challenge protocols are commonly used to assess acute allergic responses in murine AR models, whereas prolonged repeated challenges over several weeks are used to model more persistent inflammation and histopathological changes (71, 72). In our study, mice received IP OVA sensitization once weekly for 3 weeks, followed by either a 7-day INS OVA challenge for the short-term AR model, which was used in most experiments, or a 21-day INS OVA challenge for the prolonged AR model to assess histopathological changes characteristic of chronic AR (**Figure 1**).

Prior to the main OVA-AR experiments, we conducted a preliminary histamine-induced acute nasal rubbing test to determine whether electrical stimulation was necessary for the anti-pruritic effect, or whether needle insertion alone was sufficient to justify EA as the intervention modality. Histamine challenge markedly increased nasal rubbing compared with naive mice (27.00 ± 3.00 vs. 13.83 ± 1.78 bouts/10 min; p<0.01). EA at LI4–LI11 significantly attenuated this response (12.83 ± 2.54 bouts/10 min; p <0.01), whereas needling without electrical stimulation produced only a partial, non-significant reduction (18.83 ± 2.65 bouts/10 min; p = 0.0974). Based on these findings, EA was selected as the intervention modality for all subsequent experiments (**Supplementary Figure E2**).

**Figure 2:**
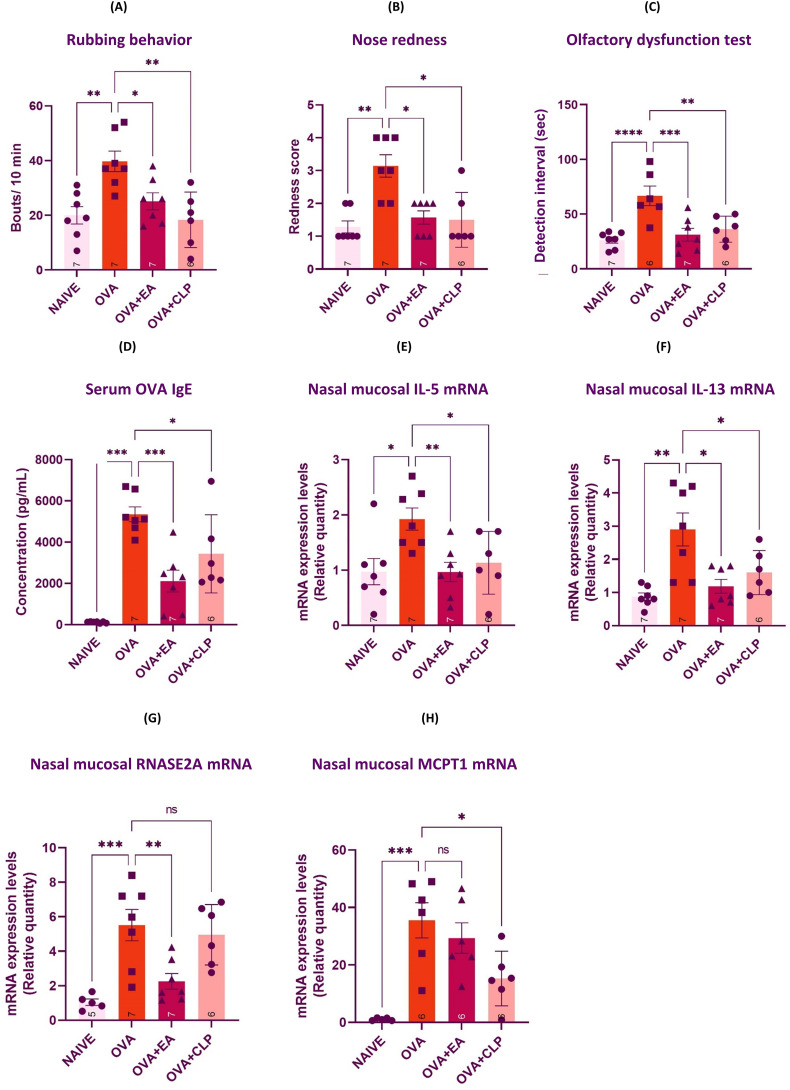
EA and CLP alleviated AR-like symptoms and modulated Th2 inflammatory and cellular biomarkers in the short-term AR model. Mice were evaluated after a 7-day OVA INS challenge with concurrent daily treatments. **(A-C)** Clinical symptom evaluations including **(A)** nasal rubbing behavior (bouts per 10 min), **(B)** nose redness visual scoring, and **(C)** olfactory dysfunction (detection interval in sec). **(D)** Systemic allergic response measured by serum OVA-specific IgE levels (pg/mL). **(E-H)** Local nasal mucosa mRNA expression assessed via RT-qPCR for **(E)** IL-5, **(F)** IL-13, **(G)** RNASE2A (eosinophil activation marker), and **(H)** MCPT1 (mast cell activation marker). The statistical was assessed via Ordinary One-way ANOVA, followed by the Holm-Šídák post hoc test in **(A, C–F)** Redness scores in panel B were analyzed using the Kruskal-Wallis test with Dunn’s post hoc test. Data are presented as mean ± standard error of the mean (SEM), n = 5–7 mice/group. Significance levels are indicated as P < 0.05 (*), P < 0.01 (**), P < 0.001 (***), and P < 0.0001 (****). Sample sizes are noted below each bar.

In the short-term AR model, nasal rubbing was 20.0 ± 3.2 bouts in naive mice and increased to 39.7 ± 3.8 bouts in OVA mice (p < 0.01). EA and the antihistamine CLP significantly reduced nasal rubbing to 25.1 ± 3.1 (p < 0.05) and 18.3 ± 4.1 bouts (p < 0.01), respectively (**Figure 2A**).

Nasal redness scores increased from 1.3 ± 0.2 in naive mice to 3.1 ± 0.3 in OVA-treated mice (p<0.01). Both EA and CLP effectively reduced redness scores to 1.6 ± 0.2 and 1.7 ± 0.3, respectively (p<0.05) (**Figure 2B**).

In the buried food pellet test, OVA-treated mice required longer to locate food (82.5 ± 17.4 sec) than naive mice (26.4 ± 2.8 sec, p < 0.001), while EA and CLP improved detection times to 31.3 ± 5.8 sec and 36.4 ± 4.9 sec, respectively (both p < 0.01) (**Figure 2C**).

### 3.2 EA at LI4+LI11 suppresses Th2 inflammatory mediators in OVA-induced AR, comparable to CLP

Exposure to allergens activated dendritic cells, leading to IgE production by B cells and Th2 differentiation, with subsequent IL-5 and IL-13 release (6).

OVA exposure increased serum OVA-IgE levels from 120.0 ± 13.3 pg/mL in naive mice to 5349.0 ± 361.3 pg/mL (p < 0.0001). EA and CLP reduced IgE levels to 2113.0 ± 532.5 pg/mL (p < 0.001) and 3433.0 ± 773.3 pg/mL (p < 0.05), respectively (**Figure 2D**).

Nasal IL-5 and IL-13 mRNA expression in the OVA group increased twofold (p<0.01) and threefold (p<0.05), respectively, indicating Th2-driven inflammation (**Figures 2E, F**). EA significantly reduced both IL-5 and IL-13 expression to near-baseline levels (p<0.05), CLP produced similar decreases (p<0.05).

### 3.3 Divergent effects of EA and CLP on local nasal biomarkers

To assess molecular correlates of eosinophil activity, RNASE2A mRNA expression (eosinophil-derived neurotoxin) (73, 74) and MCPT1 mRNA (mucosal mast cell protease 1) (75–81) were measured in the short-term AR model. OVA challenge increased RNASE2A expression approximately fivefold compared to naive controls (p < 0.001). EA reduced RNASE2A by about twofold (p < 0.01), whereas CLP had no significant effect (p=0.54, **Figure 2G**). OVA exposure elevated MCPT1 expression approximately 40-fold compared to naive controls (p < 0.001). Conversely, CLP treatment halved MCPT1 expression (p < 0.05), consistent with its mast cell–stabilizing effect, whereas EA did not significantly alter MCPT1 levels (p=0.3562, **Figure 2H**).

### 3.4 EA at LI4+LI11 attenuates eosinophil infiltration while CLP reduces mast cell activation in histopathology

**Figure 3:**
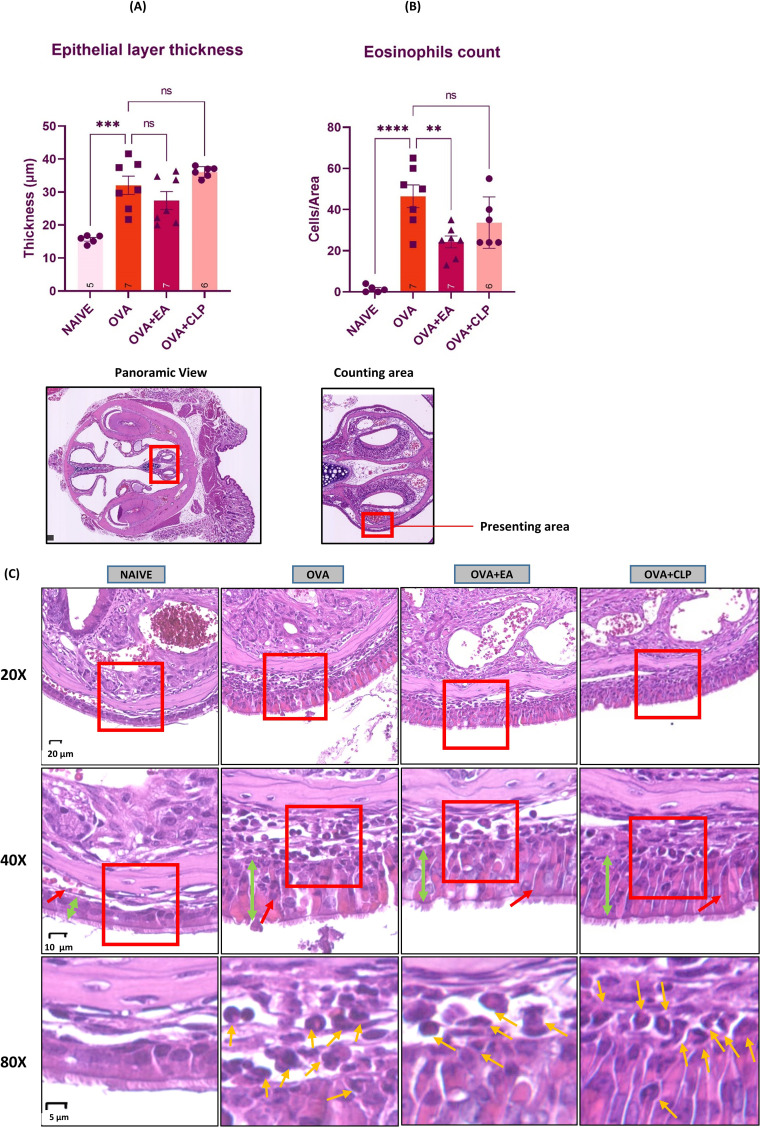
EA, but not CLP, significantly reduced eosinophil infiltration in the prolonged AR model. Histological evaluation of nasal mucosa following a 21-day OVA challenge. Bar graphs present quantitative morphometric analysis derived from the representative micrographs, showing **(A)** epithelial layer thickness (um) and **(B)** the number of infiltrated eosinophils per defined area. **(C)** Representative H&E stained sections of the nasal cavity at 20X, 40X, and 80X magnifications. Red boxes in the 20X and 40X images indicate the magnified regions of interest. Green double-headed arrows indicate the thickness of the epithelial layer. Red arrows point to standard epithelial cells, while yellow arrows in the 80X panels highlight eosinophils characterized by their bilobed nuclei and eosinophilic cytoplasm. The statistical was assessed via Ordinary One-way ANOVA, followed by the Holm-Šídák post hoc test. Data are presented as mean ± standard error of the mean (SEM), n = 5–7 mice/group. Significance levels are indicated as P < 0.01 (**), P < 0.001 (***), and P < 0.0001 (****).

In chronic allergic rhinitis, cell infiltration and tissue remodeling increase with prolonged allergen exposure (82). In the prolonged AR model, OVA mice displayed pronounced epithelial hypertrophy, with mucosal thickness increasing from 15.7 ± 0.6 µm in naive mice to 32.1 ± 2.8 µm (p < 0.001). Eosinophil infiltration increased from 1.4 ± 0.7 to 46.4 ± 5.6 cells (p < 0.0001). EA significantly reduced eosinophil counts to 24.3 ± 7.7 (p < 0.01) but did not significantly alter epithelial thickness (27.5 ± 2.7 µm, p=0.26). CLP did not significantly change either parameter, with epithelial thickness of 36.1 ± 0.7 (p=0.26), and eosinophil counts: 33.7 ± 5.1 (p=0.09, **Figures 3A–C**). In the short-term AR model, EA similarly reduced eosinophil counts compared with OVA group (**Supplementary Figure E3**), whereas CLP did not reduce eosinophil infiltration (**Supplementary Figure E3**).

**Figure 4:**
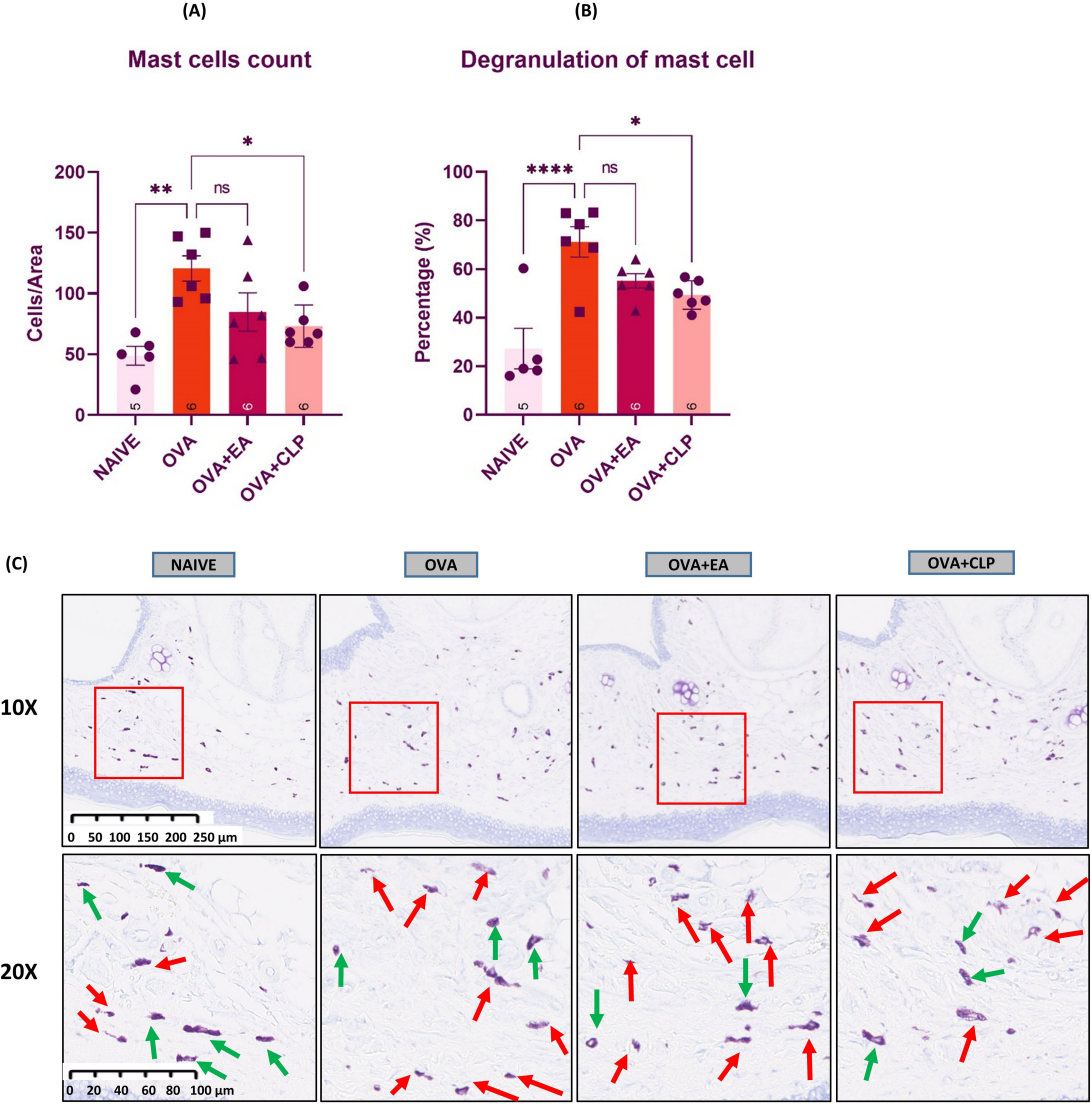
CLP, but not EA, significantly reduced mast cell infiltration and degranulation in the prolonged AR model. Histological evaluation of mast cell activity in the nasal mucosa following a 28-day OVA challenge. Bar graphs present quantitative analysis derived from the micrographs, showing **(A)** the total count of mast cells per area and **(B)** the percentage of degranulated mast cells relative to the total mast cell count. **(C)** Representative Toluidine blue-stained sections at 10X and 20X magnifications used to visualize mast cells. Red boxes in the 10X images indicate the magnified regions shown in the 20X panels. Green arrows identify intact mast cells, whereas red arrows highlight degranulated mast cells exhibiting extruded granular contents. The statistical was assessed via Ordinary One-way ANOVA, followed by the Holm-Šídák post hoc test. Data are presented as mean ± standard error of the mean (SEM), n = 5–7 mice/group. Significance levels are indicated as P < 0.05 (*), P < 0.01 (**), P < 0.001 (***), P < 0.0001 (****) and nonsignificant (ns).

Mast cell numbers in the nasal mucosa increased from 48.8 ± 7.4 in naive mice to 120.7 ± 10.4 (p < 0.01), and the degranulation rate rose from 27.3 ± 8.3% to 71.2 ± 6.3% (p < 0.0001). EA did not significantly reduce mast cell count (84.8 ± 15.7, p=0.06) and the degranulation rate (55.2 ± 3.0%, p=0.08), whereas CLP significantly reduced mast cell numbers to 73.2 ± 7.1 (p < 0.05) and the degranulation rate to 49.4 ± 2.4% (p < 0.05) (**Figures 4A–C**). Supportive 7-day histological imaging (n = 2 per group) indicated reduced mast cell activation with CLP but not EA, in line with chronic model results (**Supplementary Figure E4**).

### 3.5 The DRD1 antagonist did not reverse the effectiveness of EA

**Figure 5:**
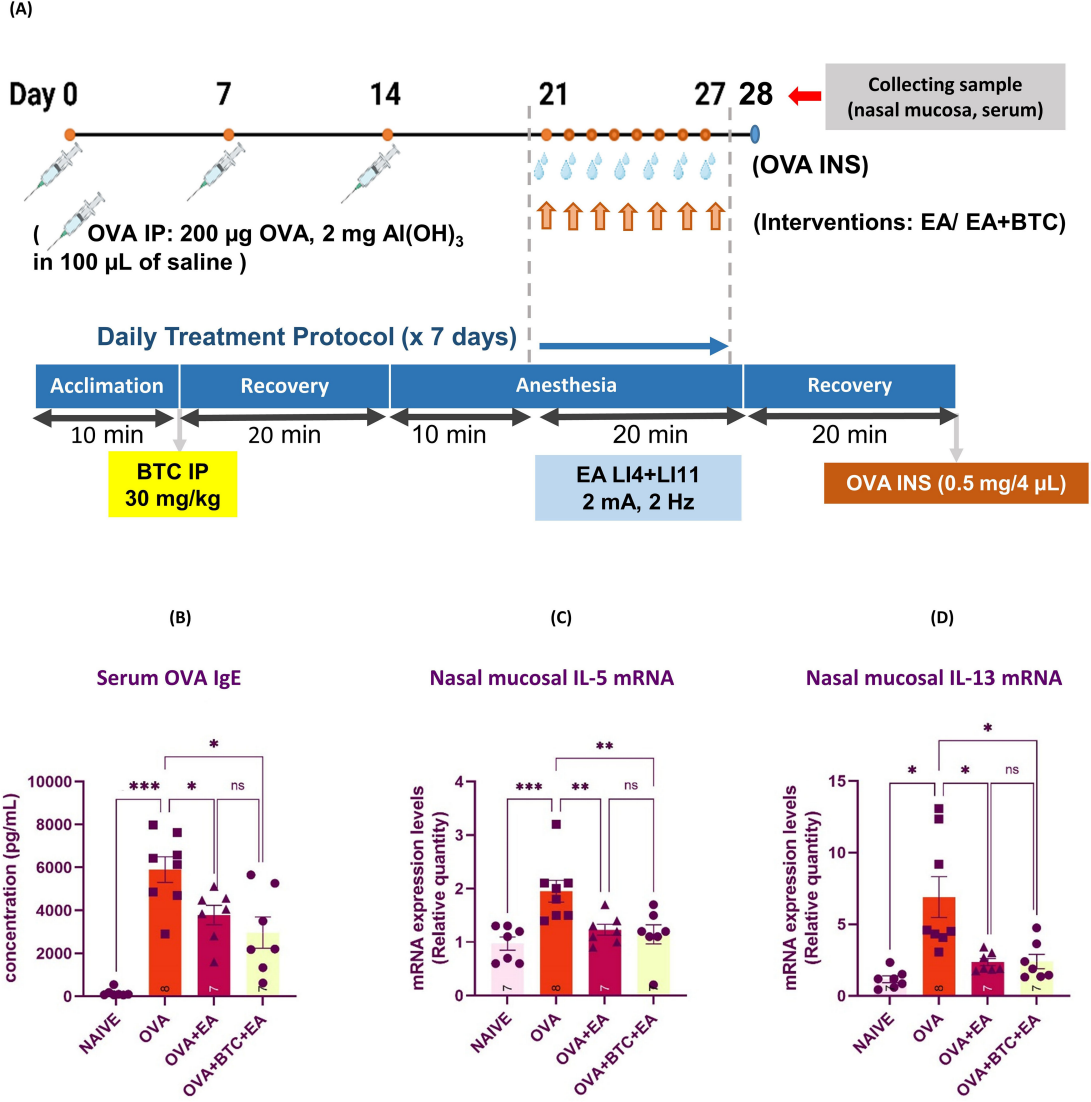
Pretreatment with a dopamine D1 receptor antagonist (butaclamol, BTC) did not reverse the EA-mediated suppression of systemic and local Th2 biomarkers in the short-term AR model. **(A)** Schematic of the 7-day OVA INS challenge and daily treatment timeline. All mice first underwent a 10-min environmental acclimation. Following this, mice in the antagonist group (OVA+BTC+EA) received an IP injection of BTC; 30 mg/kg, followed by a 20-min recovery and drug-absorption period. Mice in the other groups remained undisturbed in their cages during this interval. Subsequently, the 20-min EA session (LI4 + LI11; 2 mA, 2 Hz) was administered to the designated EA groups. Following another 20-min rest period, the OVA challenge (0.5 mg/4 µL) was administered intranasally to all groups. Bottom panels show the effects of BTC pretreatment on **(B)** serum OVA-specific IgE concentrations (pg/mL) measured via ELISA, and the relative mRNA expression levels of **(C)** IL-5 and **(D)** IL-13 in the nasal mucosa measured via RT-qPCR. The statistical was assessed via Ordinary One-way ANOVA, followed by the Holm-Šídák post hoc test. Data are presented as mean ± standard error of the mean (SEM), n = 7–8 mice/group. Significance levels indicated as P < 0.05 (*), P < 0.01 (**), P < 0.001 (***), and nonsignificant (ns).

Previous studies have demonstrated that EA at ST36 activates the sciatic and vagus nerves, promoting adrenal release of catecholamines—including dopamine (DA), EPI, NE—which in turn suppresses systemic inflammation in a lipopolysaccharide-induced mouse model (40). Given that dopamine signaling has also been implicated in EA’s analgesic and anti-inflammatory effects in neuropathic pain models (83, 84), we investigated whether dopamine D1 receptor (DRD1) activation contributes to the anti-inflammatory effects of EA in AR using the short-term AR model. To test this, mice were pretreated with butaclamol (BTC), a DRD1 antagonist, via IP injection 30 min prior to each daily EA application (**Figure 5A**).

As previously observed, the 7-day OVA challenge significantly increased OVA-IgE levels from 160.6 ± 65.2 pg/mL in naive mice to 5900.0 ± 52.2 pg/mL (p < 0.001), doubled IL-5 mRNA expression (p < 0.001), and elevated IL-13 mRNA sixfold (p < 0.001) (**Figures 5B–D**). Daily EA effectively reduced OVA-IgE to 3782.0 ± 454.4 pg/mL (p < 0.05) and halved IL-5 and IL-13 expression (both p < 0.01). Notably, BTC pretreatment did not attenuate these EA-mediated benefits; OVA-IgE levels remained reduced to 2966 ± 725.6 pg/mL (p < 0.01 vs. OVA) and suppression of IL-5 and IL-13 was sustained (both p < 0.01 vs OVA). There were no statistically significant differences between the OVA+EA group and the OVA+BTC+EA group across any of these measures (p=0.29 for OVA-IgE, p=0.72 for IL-5, p=0.97 for IL-13).

### 3.6 β_2_-AdR antagonist reversed the effectiveness of EA in AR

**Figure 6:**
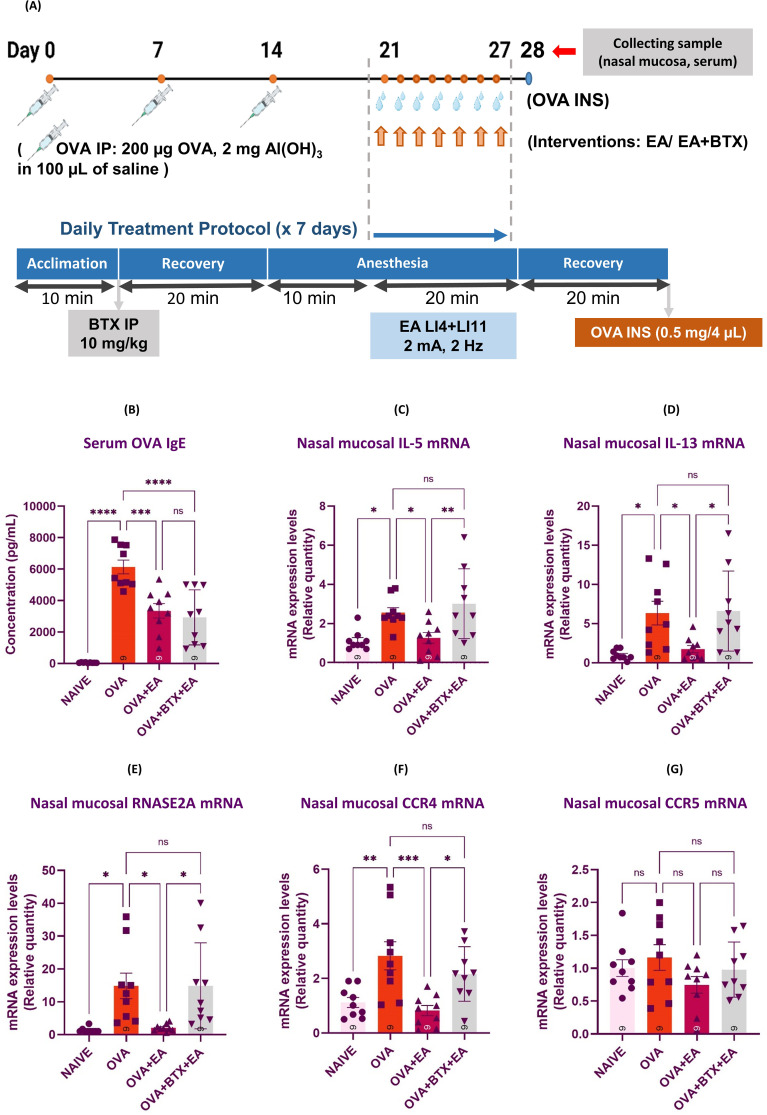
Pretreatment with a β_2_-adrenergic receptor antagonist (butoxamine, BTX) reverses EA-mediated suppression of local Th2 and eosinophil biomarkers, but not systemic IgE, in the short-term AR model. **(A)** Schematic of the 7-day OVA INS challenge and daily treatment protocol. All mice first underwent a 10-min environmental acclimation. Following this, mice in the antagonist group (OVA+BTX+EA) received an IP injection of BTX; 10 mg/kg, followed by a 20-min recovery and drug-absorption period. Mice in the other groups remained undisturbed in their cages during this interval. Subsequently, the 20-min EA session (LI4 + LI11; 2 mA, 2 Hz) was administered to the designated EA groups. Following another 20-min rest period, the OVA challenge (0.5 mg/4 µL) was administered intranasally to all groups. Bottom panels show the specific effects of BTX pretreatment on **(B)** systemic serum OVA-specific IgE concentrations (pg/mL) measured via ELISA, and the relative nasal mucosa mRNA expression levels of **(C)** IL-5, **(D)** IL-13, **(E)** RNASE2A (eosinophil marker), **(F)** CCR4 (Th2 cell marker), and **(G)** CCR5 (Th1 cell marker) measured via RT-qPCR. The statistical was assessed via Ordinary One-way ANOVA, followed by the Holm-Šídák post hoc test. Data are presented as mean ± standard error of the mean (SEM), n = 9 mice/group. Significance levels indicated as P < 0.05 (*), P < 0.01 (**), P < 0.001 (***), P < 0.0001 (****), and nonsignificant (ns).

Recent studies suggest that β_2_-AdR activation reduces eosinophil activity (52, 53). To test if β_2_-AdR activity mediates EA’s anti-inflammatory effects via NE and EPI signaling, mice were pretreated with butoxamine (BTX), a β_2_-AdR antagonist, 30 min before daily EA application in the short-term AR model (**Figure 6A**).

Systemically, OVA challenge significantly increased OVA-IgE levels to 6144.0 ± 436.7 pg/mL (p < 0.0001 vs. naive). EA reduced IgE to 3350.0 ± 462.3 pg/mL (p < 0.001 vs. OVA), while BTX+EA produced a similar reduction (2935.0 ± 285.4 pg/mL, p < 0.0001 vs. OVA, p=0.5 vs OVA+EA) (**Figure 6B**).

Locally, however, BTX pretreatment completely abolished EA’s suppression of nasal mucosal cytokines and cellular biomarkers. EA effectively reduced the elevated mRNA expression of IL-5 (p < 0.05), IL-13 (p < 0.05), and the eosinophil activation marker RNASE2A (p < 0.05) compared to the OVA group. Pretreatment with BTX reversed these benefits, restoring the expression of IL-5 (p < 0.01 vs. EA), IL-13 (p < 0.05 vs. EA), and RNASE2A (p < 0.05 vs. EA) back to or slightly above OVA-challenged levels (**Figures 6C–E**).

Furthermore, we evaluated the mRNA expression of CCR4 (a Th2 cell biomarker) and CCR5 (a Th1 cell biomarker). CCR4 expression increased nearly 3-fold with OVA challenge (p < 0.01 vs. naive) and was robustly suppressed by EA (p < 0.001 vs. OVA). BTX pretreatment significantly reversed this EA-mediated suppression, restoring CCR4 expression (p < 0.05 vs. EA) (**Figure 6F**). CCR5 expression remained unchanged across all experimental groups (p=0.63 in OVA vs Naive, p=0.21 in OVA vs OVA+EA, p=0.63 in OVA vs OVA+BTX+EA, p=0.63 in OVA+EA vs OVA+BTX+EA), confirming the specific modulation of the Th2 pathway (**Figure 6G**).

### 3.7 EA stimulated the plasma concentrations of epinephrine and norepinephrine

**Figure 7:**
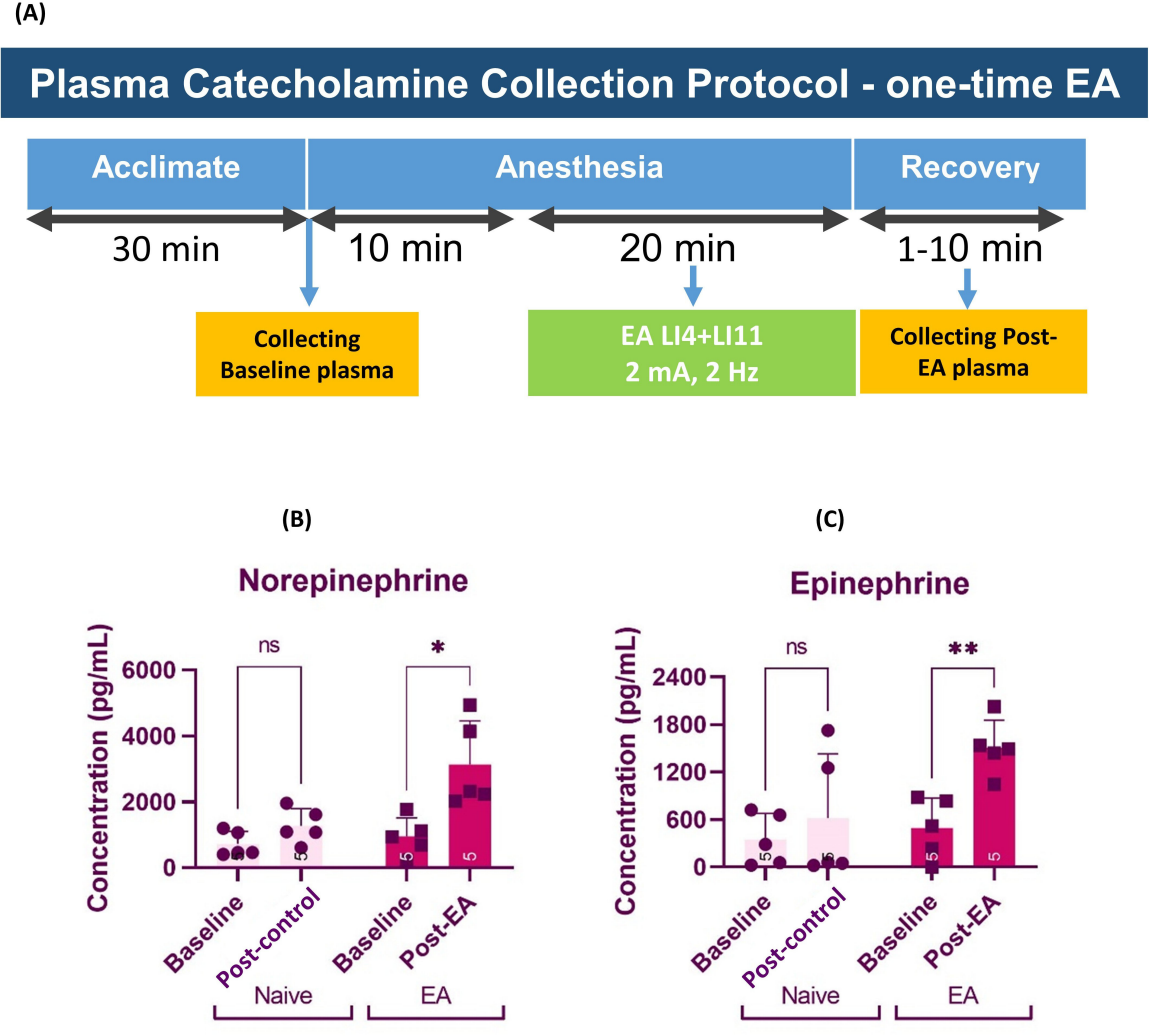
A single session of EA significantly elevated plasma concentrations of norepinephrine (NE) and epinephrine (EPI). **(A)** Schematic representation of the plasma collection protocol for the one-time EA intervention. Mice underwent a 30-min acclimation period followed by anesthesia. Baseline plasma samples were collected 10 min post-anesthesia induction. Mice in the EA group then received a 20-min EA session (LI4 + LI11; 2 mA, 2 Hz), whereas naive controls remained under anesthesia for a matched interval without needle insertion or electrical stimulation. The second plasma sample was collected within a 10-min recovery window after the intervention period. Systemic plasma concentrations (pg/mL) of **(B)** norepinephrine and **(C)** epinephrine were measured by ELISA at baseline and after the intervention period. Data demonstrate a selective modulatory effect of EA on sympathetic nervous system catecholamine release. The statistical was assessed via two-way ANOVA, followed by the Šídák post hoc test. Data are presented as mean ± standard error of the mean (SEM), n = 5 mice/group, with significance levels indicated by p < 0.05 (*) and p < 0.01 (**).

Having established that β_2_-AdR signaling is required for EA’s local anti-inflammatory effects in the nasal mucosa, we next investigated whether EA actively stimulates the systemic release of endogenous catecholamines. Plasma NE and EPI levels were measured by ELISA at baseline and after a matched control interval in naive mice or after a single EA session in the EA group. To ensure accuracy, baseline plasma was collected 10 min after the induction of anesthesia. The second plasma sample was collected after a matched anesthesia/control interval in naive mice and within 1–10 min after completion of the 20-min EA treatment in the EA group (**Figure 7A**).

As shown in **Figure 7B**, NE levels in naive mice remained stable after the matched control interval (720.7 ± 170.3–1269.2 ± 234.9 pg/mL, p=0.37), but in the EA group, NE increased more than threefold from 949.6 ± 253.1 to 3132.8 ± 591.9 pg/mL (p<0.05) post-EA. Similarly, in **Figure 7C**, EPI levels changed minimally in naive mice (349.8 ± 146.2–622.0 ± 361.8 pg/mL, p=0.20), while in the EA group, EPI increased significantly from 494.1 ± 169.4 to 1507.0 ± 155.7 pg/mL (p<0.01). Two-way ANOVA confirmed a significant interaction between group and time (p < 0.05). *Post hoc* analysis showed no significant difference in baseline concentrations between groups (p = 0.88 for both NE and EPI), whereas after the intervention interval, the catecholamine changes in the EA group were significantly greater than those in the naive group (p < 0.01 for NE and p < 0.05 for EPI).

### 3.8 EA-mediated relief of AR-like behavior and eosinophil infiltration is reversed by β_2_-AdR antagonist and mimicked by exogenous EPI

**Figure 8:**
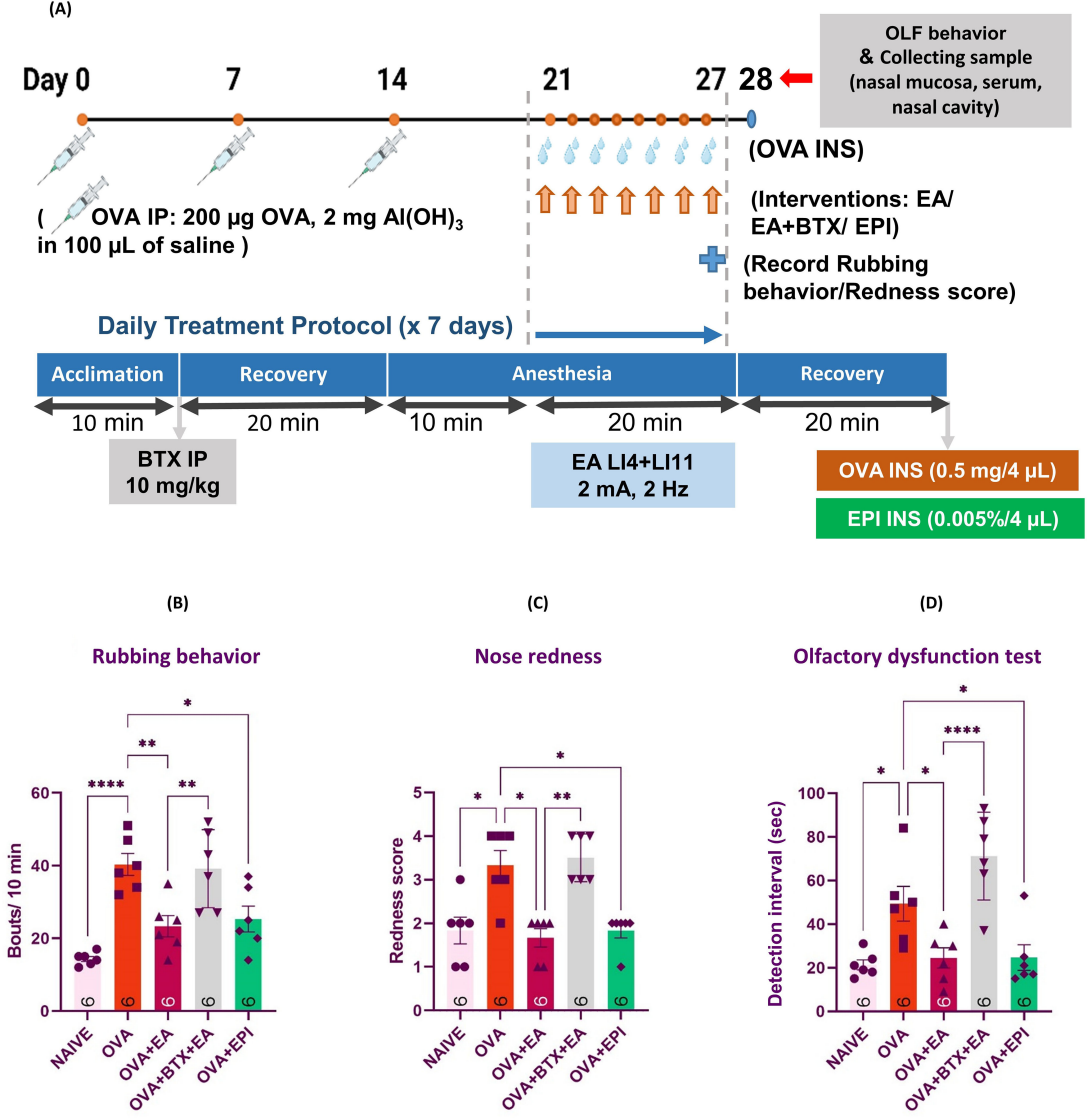
The β_2_-adrenergic antagonist (butoxamine, BTX) reversed, and exogenous EPI mimicked, the EA-mediated alleviation of clinical symptoms in the short-term AR model. **(A)** Timeline and daily treatment protocol for the 7-day OVA INS challenge. All mice first underwent a 10-min environmental acclimation. Following this, mice in the antagonist group (OVA+BTX+EA) received an IP injection of BTX; 10 mg/kg, followed by a 20-min recovery and drug-absorption period. Mice in the other groups remained undisturbed in their cages during this interval. Subsequently, the 20-min EA session (LI4 + LI11; 2 mA, 2 Hz) was administered to the designated EA groups. Following another 20-min rest period, the OVA challenge (0.5 mg/4 µL) was administered intranasally to all groups. Finally, mice in the OVA+EPI group received 0.005% EPI via INS drip (4 μL/mouse) 3 min after their daily OVA challenge. Clinical symptom evaluations including **(B)** nasal rubbing behavior (bouts per 10 min), **(C)** nose redness visual scoring, and **(D)** olfactory dysfunction (detection interval in sec). BTX pretreatment was used to determine if β_2_-AdR blockade reverses the beneficial effects of EA, while INS EPI was used to determine if direct receptor activation mimics the effects of EA. The statistical was assessed via Ordinary One-way ANOVA, followed by the Holm-Šídák post hoc test. Data are presented as mean ± standard error of the mean (SEM), n = 6 mice/group. Significance levels are indicated as P < 0.05 (*), P < 0.01 (**), P < 0.001 (***), and P < 0.0001 (****).

Given that epinephrine has a higher binding affinity for β_2_-AdR, while NE more commonly activates α_1_-AdR (85), we focused on EPI to further explore EA’s mechanism. To test whether the anti-inflammatory effects of EA are mediated through β_2_-AdR signaling, we compared the responses of daily EA-treated, EA+BTX, and EPI-treated mice in a 7-day OVA-induced short-term AR model (**Figure 8A**).

In behavioral testing, nasal rubbing increased from 14.3 ± 0.7 bouts in naive mice to 41.2 ± 3.0 bouts after OVA (p < 0.0001). EA reduced rubbing to 23.3 ± 2.9 bouts (p < 0.01 vs. OVA), an effect abolished by BTX (39.2 ± 4.4 bouts, p < 0.01 vs. EA). EPI treatment mimicked EA, reducing rubbing to 25.3 ± 3.6 bouts (p < 0.01 vs. OVA) (**Figure 8B**). Nasal redness scores increased from 1.8 ± 0.3 in naive mice, to 3.3 ± 0.3 in OVA mice (p < 0.01) and were reduced by EA (1.9 ± 0.2) and EPI (1.8 ± 0.2) (both p < 0.05 vs. OVA), but not by BTX+EA (3.5 ± 0.2, p < 0.01 vs. EA) (**Figure 8C**).

In the olfactory test, OVA mice required 49.3 ± 8.0 sec to detect food vs. 21.3 ± 2.9 sec in naive mice (p < 0.05). EA improved detection to 24.5 ± 4.7 sec (p < 0.05 vs. OVA), but this effect was blocked by BTX+EA (71.2 ± 8.2 sec, p < 0.001 vs. EA). EPI produced similar improvement (24.7 ± 5.9 sec, p < 0.05 vs. OVA) (**Figure 8D**).

**Figure 9:**
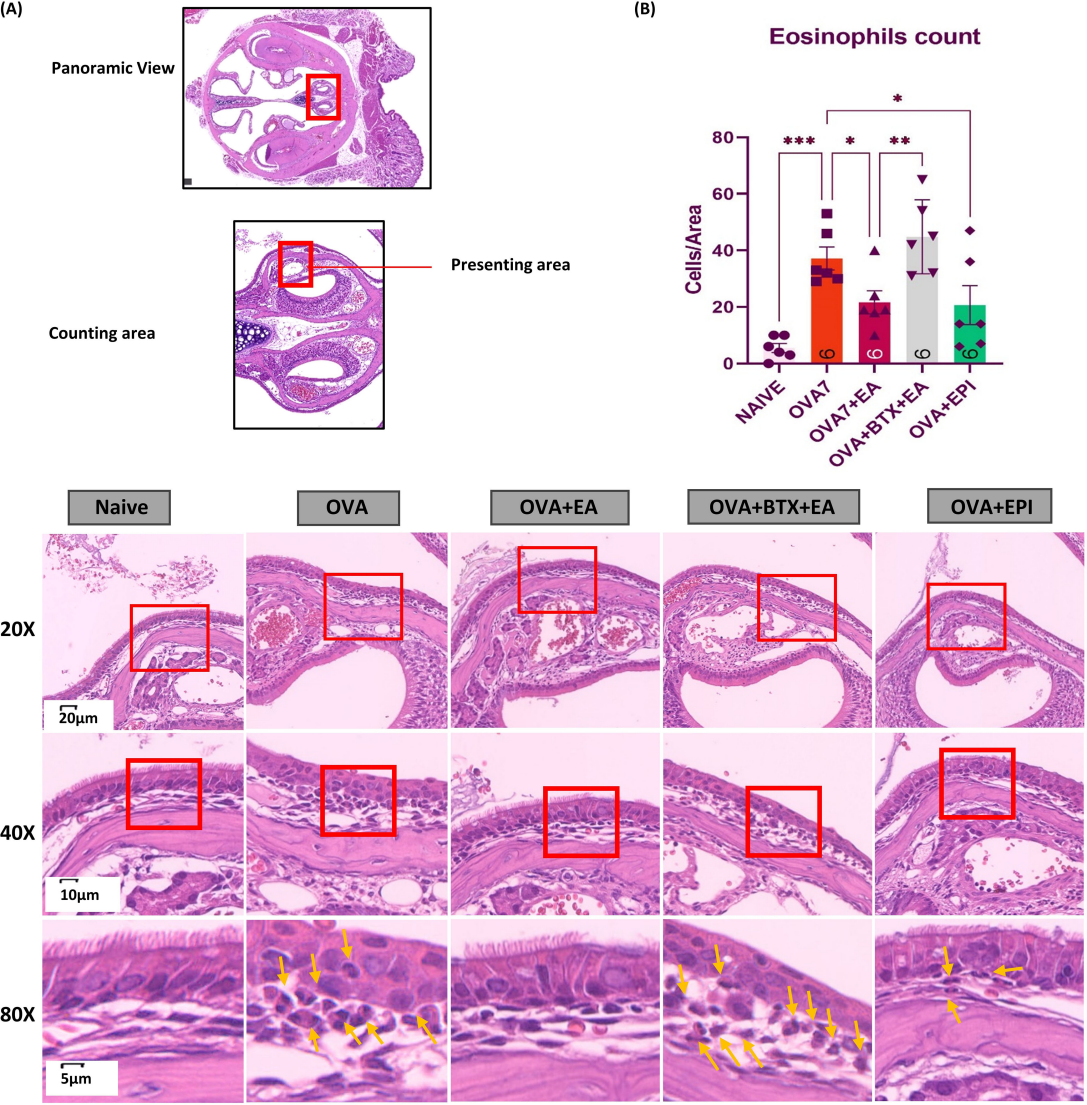
The β_2_-adrenergic antagonist reversed and exogenous EPI mimicked, EA-mediated suppression of eosinophil infiltration in the short-term AR model. **(A)** H&E stained sections of the nasal mucosa across treatment groups at 20X, 40X, and 80X magnifications. Red boxes in the 20X and 40X images define the magnified regions of interest. Yellow arrows in the 80X panels identify infiltrating eosinophils within the disrupted mucosal architecture. **(B)** Quantitative morphometric analysis of eosinophil counts per defined area. BTX pretreatment successfully reversed the EA-induced reduction in eosinophils, whereas EPI administration replicated the EA-induced reduction. The statistical was assessed via Ordinary One-way ANOVA, followed by the Holm-Šídák post hoc test. Data are presented as mean ± standard error of the mean (SEM), n = 6 mice/group. Significance levels are indicated as P < 0.05 (*), P < 0.01 (**), and P < 0.001 (***).

Histology showed eosinophil counts increased from 5.5 ± 1.6 cells/area in naive mice to 37.2 ± 4.0 with OVA (p < 0.0001). EA robustly reduced eosinophil counts to 21.7 ± 4.1 (p < 0.001 vs. OVA). Pretreatment with BTX completely reversed this anti-inflammatory effect, restoring high levels of eosinophil infiltration (44.8 ± 5.3, p < 0.01 vs. EA). Conversely, exogenous EPI treatment effectively mimicked EA, significantly reducing eosinophil counts to 20.7 ± 6.9 (p < 0.001 vs. OVA) (**Figure 9**).

## 4 Discussion

**Figure 10:**
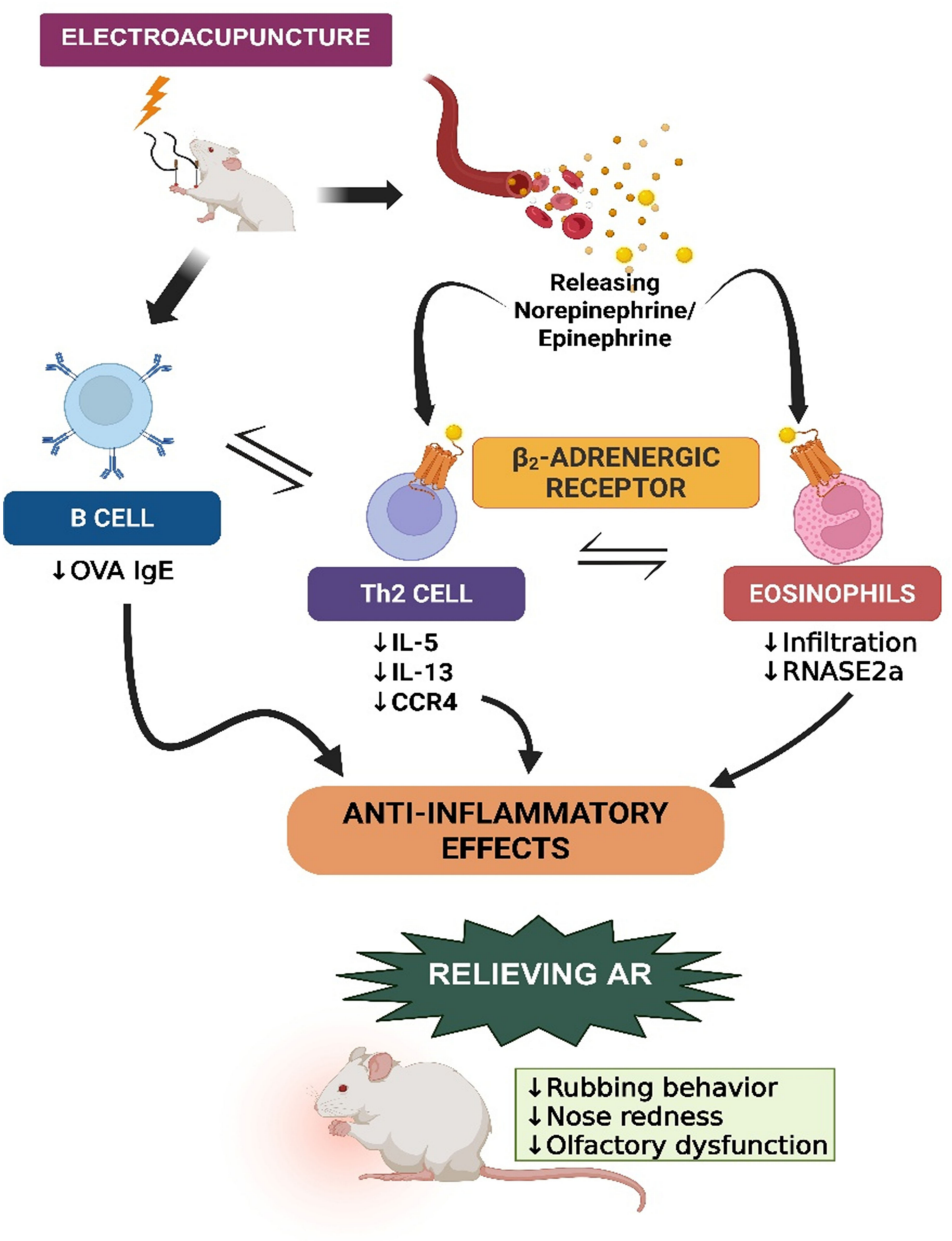
Proposed pathway by which electroacupuncture (EA) modulated allergic rhinitis (AR) through β_2_-AdR. EA stimulated the release of norepinephrine (NE) and epinephrine (EPI) that act on β_2_-adrenergic receptors (β_2_-AdR) expressed on Th2 cells and eosinophils. β_2_-AdR activation suppressed IL-5 and IL-13 secretion from Th2 cells, thereby reducing eosinophil recruitment and activation. Direct β_2_-AdR signaling on eosinophils further decreased their infiltration and RNASE2A expression in the nasal mucosa. EA also attenuated B-cell–mediated IgE production indirectly via reduced IL-13. Collectively, these effects dampened airway inflammation, alleviating AR symptoms such as nasal rubbing and olfactory dysfunction.

In this study, we present novel evidence that EA exerts significant anti-inflammatory effects on AR by specifically targeting eosinophil infiltration via the β_2_-AdR pathway (**Figure 10**). We hypothesize that EA triggers the release of NE and EPI into the plasma, which act on β_2_-AdR located on Th2 cells and eosinophils. Activation of β_2_-AdR on Th2 cells suppresses the production of proinflammatory cytokines IL-5 and IL-13, thereby reducing eosinophil infiltration and activity. Furthermore, activation of β_2_-AdR on eosinophils directly inhibits their activity, reducing their presence in the nasal mucosa. Consequently, EA alleviates AR-related behaviors: rubbing, nose redness, and olfactory dysfunction.

### 4.1 β_2_-AdR is essential for the anti-inflammatory effect of EA

The sympathetic nervous system plays a key role in EA’s anti-inflammatory effects, mediated by catecholamines such as DA, NE, EPI (84, 86–92). Our study is the first to demonstrate that β_2_-AdR activation is required for EA’s anti-inflammatory effects in AR, wherein EA stimulates sympathetic release of NE and EPI to mediate this response. In the OVA-induced AR model, EA alleviated nasal symptoms (rubbing, redness, and olfactory dysfunction) and reduced Th2-associated inflammatory markers, including OVA-IgE, IL-5, IL-13 mRNA, RNASE2A, CCR4, and eosinophil infiltration. All reductions—except for OVA-IgE—were abolished by β_2_-AdR blockade, whereas DRD1 blockade had no effect.

Allergen exposure disturbs catecholamine homeostasis. NE and EPI initially rise in response to allergens but decline with chronic inflammation, likely due to overstimulation (41, 43–45). This reduction may lead to the impairment of balancing T cell activities. In the recent report, NE, through β_2_-AdR, can inhibit the shift of CD4+ T cells toward Th17 cells (51). Moreover, altered adrenoceptor profiles—including β-AdR shifts—have been observed in nasal allergy (44, 93). While its traditional known function regulates vascular tone, β_2_-AdR also modulates immune balance and leukocyte function (47–50). These physiological and clinical anchors highlight the relevance of catecholamine pathways in airway disease and provide the rationale for our novel investigation into β_2_-AdR signaling in EA-mediated AR regulation. Consistent with this role, EA decreased IL-5 and IL-13 mRNA levels, effects that were reversed by β_2_-AdR blockade, supporting a catecholamine-driven, β_2_-dependent anti-inflammatory mechanism in AR.

Although DA has been linked to EA’s effects on neuropathic pain and inflammation (83, 84), our study suggests it is not essential for EA’s efficacy. EA also lowered OVA-IgE, but this effect persisted under β_2_-AdR blockade. The relationship between catecholaminergic signaling and IgE regulation appears to be complex, with previous studies reporting mixed findings regarding adrenergic effects on B-cell IgE responses (94–96). Therefore, the reduction of OVA-specific IgE by EA in our study may involve mechanisms distinct from the β_2_-AdR-dependent local anti-inflammatory pathway identified here.

Together, these findings reveal for the first time that EA’s anti-inflammatory effects in AR are primarily mediated through catecholamine-driven β_2_-AdR signaling rather than DA or IgE-related mechanisms.

### 4.2 EA targets eosinophilic inflammation in AR via β_2_-adrenergic signaling while CLP targets mast cells

Previous studies have shown that acupuncture and related therapies (e.g., warm acupuncture, acupoint injection, herbal acupoint application) alleviate eosinophilic inflammation in allergic airway models such as asthma and AR (97–100). However, the underlying mechanism remains unclear. Our findings extend these observations by revealing that EA targets eosinophils via β_2_-AdR signaling. Independent *in vitro* studies show β_2_-AdR activation dampens eosinophil effector functions—lower adhesion, superoxide generation, and EDN release (52, 53). Together with reports of adrenoceptor abnormalities in nasal allergy (44, 93), these findings highlight β_2_-AdR as a mechanistic regulator of eosinophil-driven inflammation. Building upon this foundation, our study provides the first *in vivo* evidence that EA targets eosinophilic inflammation through β_2_-AdR signaling in AR.

Both EA and CLP reduced AR symptoms and Th2 allergic biomarkers (OVA IgE, IL-5, IL-13), but their cellular targets diverged: EA specifically decreased eosinophil infiltration and RNASE2A expression, whereas CLP mainly inhibited mast-cell activation and MCPT1 expression. Because eosinophils are pivotal drivers of Th2 inflammation (13, 14, 17), our findings newly identify EA as a modality that directly acts on the eosinophilic phase of AR pathology, in contrast to CLP, which predominantly suppresses the mast-cell phase. These results align with previous studies reporting that CLP has minimal effects on eosinophil recruitment and inflammation in AR (101); or requires high concentrations and low IL-5 conditions *in vitro* to achieve inhibition (102). Altogether, EA provides an effective non-pharmacologic approach to controlling eosinophil-dominant AR inflammation that remains insufficiently addressed by antihistamines or corticosteroids.

Collectively, this study is the first to reveal a β_2_-adrenergic–dependent, eosinophil-targeting mechanism of EA in allergic rhinitis, providing mechanistic contrast to CLP’s mast-cell–focused action and underscoring EA as an effective non-pharmacologic strategy for managing eosinophil-dominant Th2 inflammation.

### 4.3 EPI mimics EA effects and suggests a novel application for β_2_ agonists in AR

In addition, exogenous EPI reproduced EA’s improvements in AR symptoms and eosinophil infiltration, supporting the interpretation that catecholamine-β_2_-AdR signaling mediates the anti-inflammatory effects of EA on the eosinophilic axis. Clinically, EPI is conventionally used as a life-saving treatment for systemic anaphylaxis (103–105). Our findings, together with emerging evidence that intranasal EPI can relieve allergic symptoms in food-challenge patients (106), suggest the potential repurposing of β_2_-agonists or low-dose topical EPI as adjunctive treatments for eosinophilic AR while minimizing systemic effects.

Although EA increased both plasma NE and EPI, these catecholamines may reflect distinct biological sources and modes of action. NE is classically released from postganglionic sympathetic nerve terminals and may act locally within innervated tissues, whereas circulating EPI is mainly derived from adrenal medullary chromaffin cells and acts as a systemic endocrine mediator (107). In our study, the elevation of plasma NE and EPI after LI4–LI11 EA supports activation of systemic autonomic/sympatho-adrenal catecholamine signaling. Moreover, intranasal EPI reproduced EA-like improvements in AR symptoms and eosinophil infiltration, indicating that β_2_-AdR activation within the nasal inflammatory microenvironment is sufficient to mimic EA’s anti-eosinophilic effects. However, these findings do not determine whether endogenous catecholamines act through adrenal-derived circulating EPI, local nasal sympathetic NE release, or both. This question is biologically relevant given growing evidence for airway neuroimmune regulation and anatomically distinct autonomic pathways engaged by EA (108, 109). Future studies using adrenalectomy, cervical sympathectomy, local denervation, and tissue catecholamine measurements will be required to define the anatomical pathway more precisely.

Acute and chronic adrenergic activation may have different immunological consequences. In the present context, a transient EA-induced sympathoadrenal pulse may promote β_2_-AdR engagement and contribute to anti-inflammatory signaling, whereas chronic or repeated stress exposure has been associated with receptor desensitization, impaired adrenergic regulation, and persistence of Th2-skewed inflammation (110). Thus, the catecholamine response triggered by controlled EA should not be assumed to be equivalent to chronic stress biology. Future studies should directly compare EA-induced catecholamine signaling with stress-related autonomic activation using appropriate stress biomarkers and control conditions.

### 4.4 Limitations

While our findings highlight the role of β_2_-AdR in mediating the effects of EA, further studies are needed to determine whether EA reduces IgE via alternative pathways or involves other adrenergic receptors. The long-term effects of EA on IL-5, IL-13, and RNASE2A, as well as its indirect impact on mast cells and other immune cells, remain unexplored. Additionally, the absence of catecholamine measurements in the OVA group limits the ability to directly correlate sympathetic activation with behavioral and immune changes. The additional experiment demonstrated that EA produced a significantly greater reduction in histamine-induced rubbing than needling alone, supporting an important contribution of electrical stimulation. Nevertheless, the absence of a non-acupoint sham EA group remains a limitation. Although this control was not included in the present study, previous studies have shown that limb acupoint stimulation can induce autonomic and inflammatory responses distinct from those elicited by control-site stimulation (108). Moreover, only one EA protocol was tested in this study; thus, the relative contribution of stimulation parameters such as current intensity, frequency, and duration requires further investigation. Future studies comparing non-acupoint sham EA, standardized manual acupuncture, combined therapy with CLP, and denervation or adrenalectomy approaches would help clarify acupoint specificity, stimulation dependency, and the anatomical source of catecholamine signaling. Species-specific differences, including MCPT1 expression, pose translational challenges, and the need for standardized EA protocols is crucial to ensure consistent and reproducible outcomes in both preclinical and clinical studies. Moreover, although serum OVA-specific IgE and plasma catecholamines were measured at the protein/metabolite level, local nasal cytokines and cellular biomarkers were assessed mainly at the mRNA level because of limited tissue availability; future studies should confirm these findings at the protein level. Finally, because the prolonged AR model was used primarily for histopathological assessment, future studies should also evaluate behavioral, Th2, and additional cellular biomarker outcomes in this model.

## 5 Conclusion

EA activates sympathetic catecholamine release to engage β_2_-AdR, suppressing Th2 cytokines and eosinophilic inflammation to relieve AR; and this eosinophil-targeting pathway is distinct from CLP, which primarily modulates mast-cell activity. Notably, INS EPI mimics EA’s effects, suggesting β_2_-agonists or low-dose topical EPI could be repurposed as adjunctive therapies for eosinophil-dominant AR.

## Data Availability

The data analyzed in this study is subject to the following licenses/restrictions: The datasets used and/or analyzed during the current study are available from the corresponding author upon reasonable request. Requests to access these datasets should be directed to Y-HC, yihungchen@mail.cmu.edu.tw.

## Glossary

AR: allergic rhinitis
AdR: Adrenergic receptor
β2-AdR: β2 adrenergic receptor
BTC: Butaclamol
BTX: Butoxamine
CCR4: C-C motif chemokine receptor 4
CLP: Chlorpheniramine
DRD1: dopamine D1 receptor
EA: Electroacupuncture
EDN: Eosinophil-derived neurotoxin
ELISA: Enzyme-linked immunosorbent assay
EPI: Epinephrine
HE: Hematoxylin & eosin
IL-: Interleukin
INS: Intranasal
NE: Norepinephrine
OVA: Ovalbumin
qPCR: quantitative polymerase chain reaction
RNASE2A: Ribonuclease A family member 2
TB: Toluidine blue
Th1: T helper cell type 1
Th2: T helper cell type 2
