# Supplementary material for: Electroacupuncture as an eosinophil-targeting treatment in ovalbumin-induced allergic rhinitis involving β2-adrenergic receptor in a mouse model
Source: Front Immunol. 2026 Jun 16;17:1832773. doi: 10.3389/fimmu.2026.1832773 (PMC13314418; doi:10.3389/fimmu.2026.1832773)
Supplement: Supplementary file 1 [file DataSheet1.docx]

## **Supplementary table E1: Drug, Biochemistry commercial kit, Machine and Software**

| **Category** | **Name** | **Usage** | **Brand** | **Country** |
| --- | --- | --- | --- | --- |
| **Drug/Biochemistry Kit** | Ovalbumin (OVA) | Intraperitoneal injection (100 µL saline), intranasal (4 µL, 0.5 mg OVA in distilled water) | Sigma-Aldrich | Saint Louis, MO |
|  | Butoxamine (BTX) | 10 mg/kg, intraperitoneal injection before EA | Sigma-Aldrich | Saint Louis, MO |
|  | Butaclamol (BTC) | 30 mg/kg, intraperitoneal injection before EA | Sigma-Aldrich | Saint Louis, MO |
|  | Epinephrine | 0.005%  4 μl solution each mouse | Sigma-Aldrich | Saint Louis, MO |
|  | Chlorpheniramine (CLP) | 10 mg/kg, oral gavage 20 minutes before OVA challenge | Sigma-Aldrich | Saint Louis, MO |
|  | RNAlater Stabilization Solution | Tissue preservation for RNA stabilization | Thermo Fisher Scientific | USA |
|  | PureLink® RNA Mini Kit | RNA extraction | Ambion | USA |
|  | High-Capacity cDNA Reverse Transcription Kit | cDNA synthesis | Applied Biosystems | USA |
|  | Applied Biosystems™ Fast SYBR™ Green Master Mix | qPCR amplification | Applied Biosystems | USA |
|  | ELISA Kits (Catecholamines) | Plasma NE and EPI concentration measurement | LND | Germany |
|  | OVA-IgE ELISA Kit | Specific OVA allergen IgE measurement | Cayman | USA |
| **Machines** | Ito Trio-300 Stimulator | Electronic current stimulation for electroacupuncture | Ito | Japan |
|  | NanoZoomer-XR Digital Slide Scanner | Scanning and processing histological slides | Hamamatsu | Japan |
|  | StepOnePlus™ Real-Time PCR System | Quantitative PCR analysis | Applied Biosystems | USA |
| **Software** | NDP.view2 | Viewing and analyzing digital slide images | Hamamatsu | Japan |
|  | GraphPad Prism Software 9.0 | Statistical analysis and graph generation | GraphPad Software | USA |

## **Supplementary table E2: Primers’s Sequences**

Species: Mus Muculus

Picked primers were chosen by The Basic Local Alignment Search Tool (BLAST)

| **Gene** | **Sequence** | **Sequence from 5’ to 3’ direction** |
| --- | --- | --- |
| IL-5 | Forward | TCACCGAGCTCTGTTGACAA |
|  | Reverse | CCACACTTCTCTTTTTGGCG |
| IL-13 | Forward | CCTGGCTCTTGCTTGCCTT |
|  | Reverse | GGTCTTGTGTGATGTTGCTCA |
| CCR4 | Forward | TCTACAGCGGCATCTTCTTCAT |
|  | Reverse | CAGTACGTGTGGTGGTGCTCTG |
| CCR5 | Forward | GTCTACTTTCTCTTCTGGACTCC |
|  | Reverse | CCAAGAGTCTCTGTTGCCTGCA |
| MCTP1 | Forward | CCCTAGAAGCTCACCAAGGC |
|  | Reverse | CCCCCAGAGTCTCCCATGTAT |
| RNASE2a | Forward | ACATGGGTCTGGAGCAACTT |
|  | Reverse | CATCGGGGATAGGCTCTGTT |
| GAPDH | Forward | TGTGTCCGTCGTGGATCTGA |
|  | Reverse | TTGCTGTTGAAGTCGCAGGAG |

## **Supplementary Figure E1: RGB parameter categorize for nose redness**


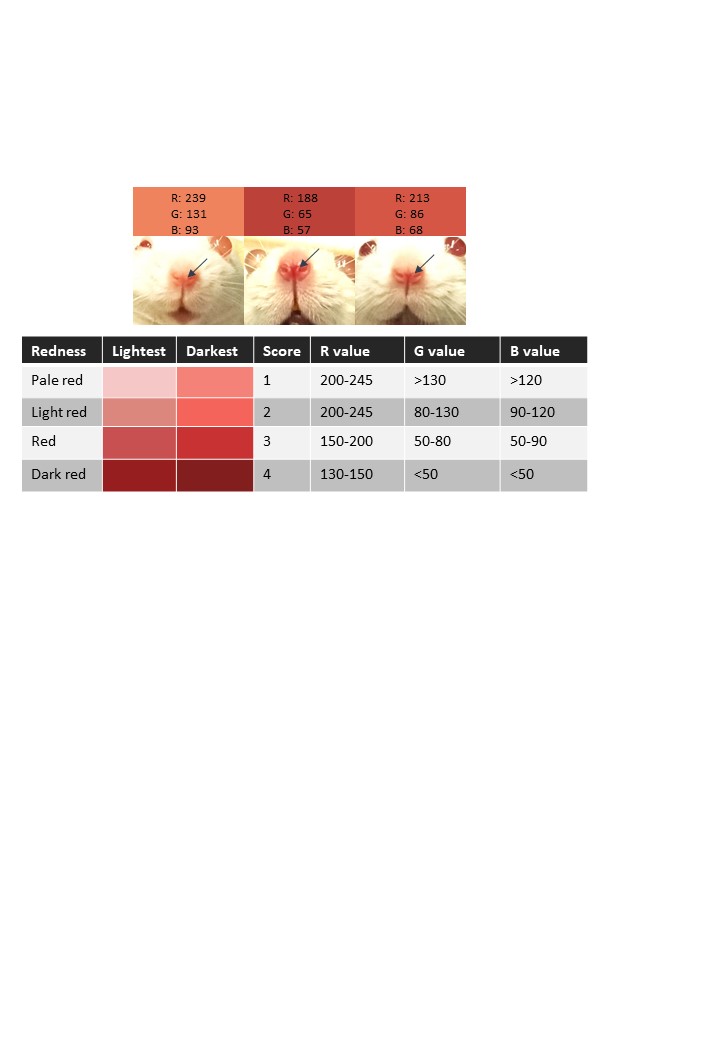


Representative images illustrating the RGB (red–green–blue) parameter-based method used to categorize nasal redness. Digital images of the nasal region were analyzed using RGB channel intensity to objectively quantify redness. Increased redness was associated with higher red-channel intensity relative to green and blue channels, enabling semi-quantitative classification of nasal inflammation severity.

**Supplementary Figure E2: Effects of LI4-LI11 electroacupuncture and needling-only intervention on histamine-induced nasal rubbing behavior**

**
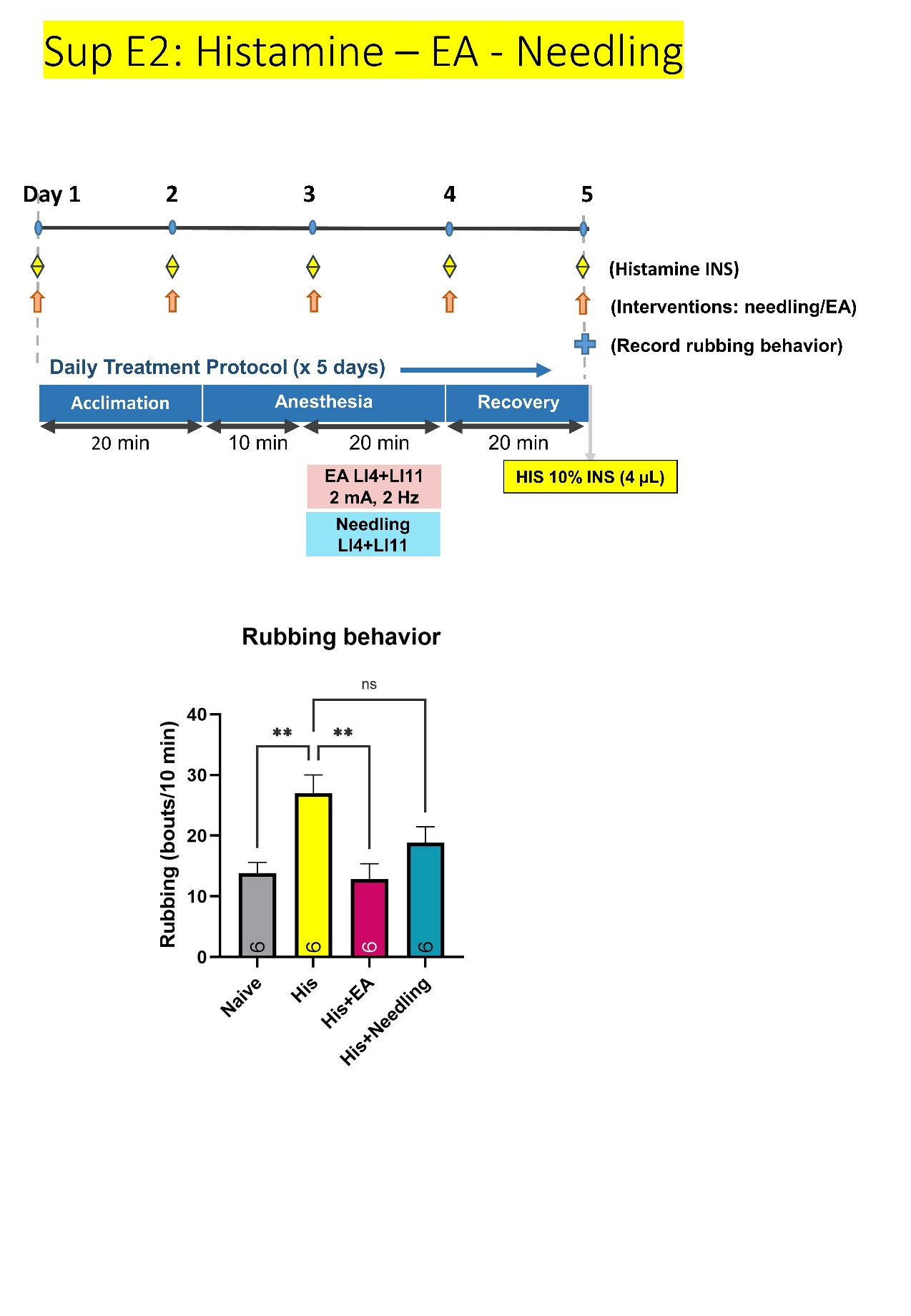
**

**(A) Schematic of the experimental design for the histamine-induced acute nasal rubbing model.** Mice received LI4-LI11 electroacupuncture (EA) or LI4-LI11 needling without electrical stimulation, followed by histamine-intranasal challenge on Days 1-5. On Day 5, mice were challenged intranasally with histamine or normal saline, and nasal rubbing behavior was recorded. **(B) Quantification of nasal rubbing behavior (bouts/10 min).** Histamine significantly increased rubbing behavior compared with the naive group. EA significantly reduced histamine-induced rubbing, whereas LI4-LI11 needling without electrical stimulation did not significantly reduce rubbing. The statistical was assessed via Ordinary One-way ANOVA, followed by the Sidak post hoc test. Data are presented as mean ± SEM, n = 6 mice/group. Significance levels are indicated as P < 0.01 (**).

**Supplementary Figure E3: EA at LI4+LI11 Attenuates Eosinophil Infiltration While CLP Shows Minimal Effect 7-day OVA model**

##
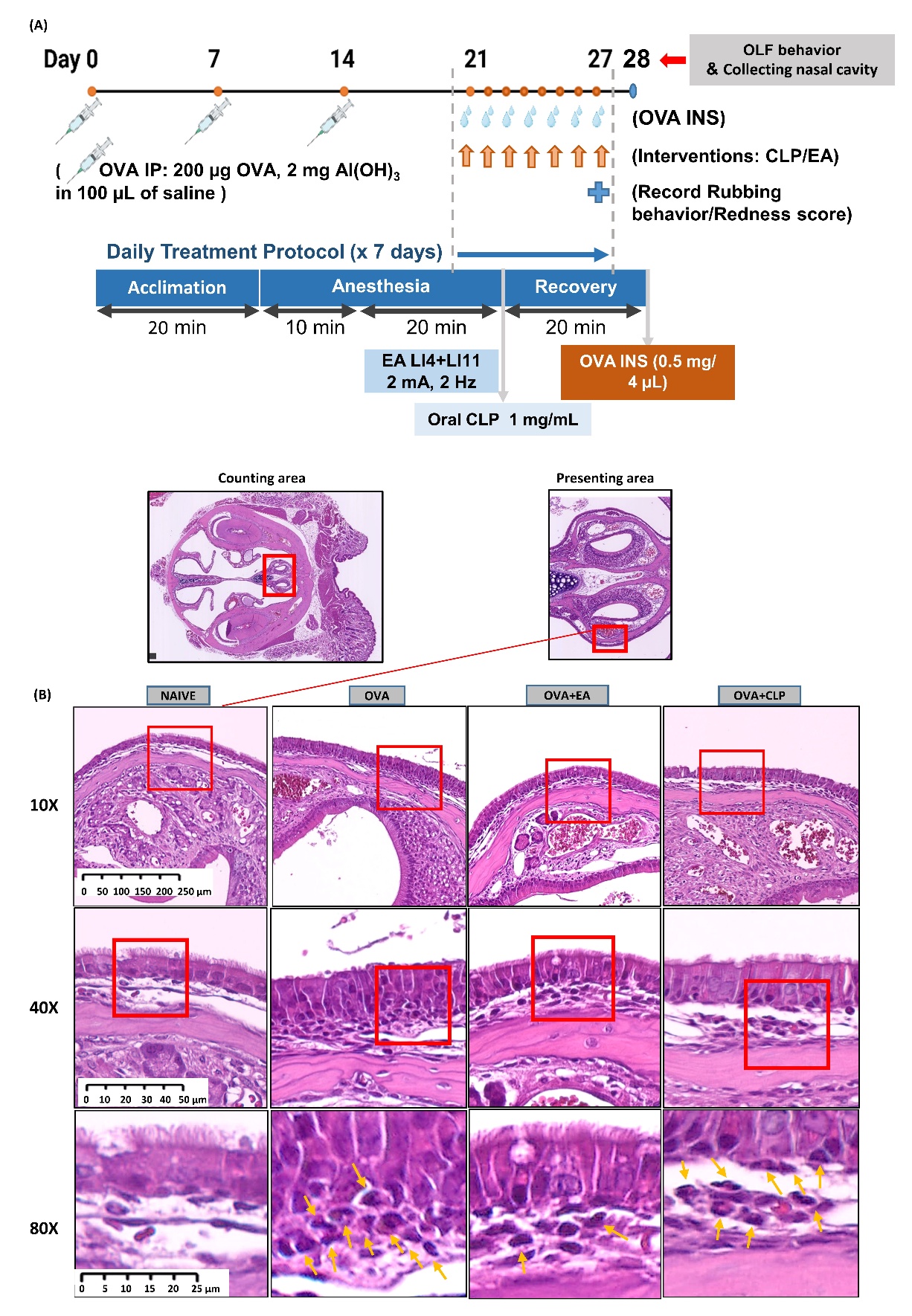


Representative histological images and quantification of eosinophil infiltration in nasal mucosal tissue following 7-day OVA challenge. OVA significantly increased eosinophil infiltration compared with naive controls. Electroacupuncture (EA) at LI4-LI11 markedly reduced eosinophil infiltration, whereas chlorpheniramine (CLP) showed minimal effect (n = 2 mice/group).

**Supplementary Figure E4: Only CLP decreased Mast cells number and degranulation in 7-day OVA model**


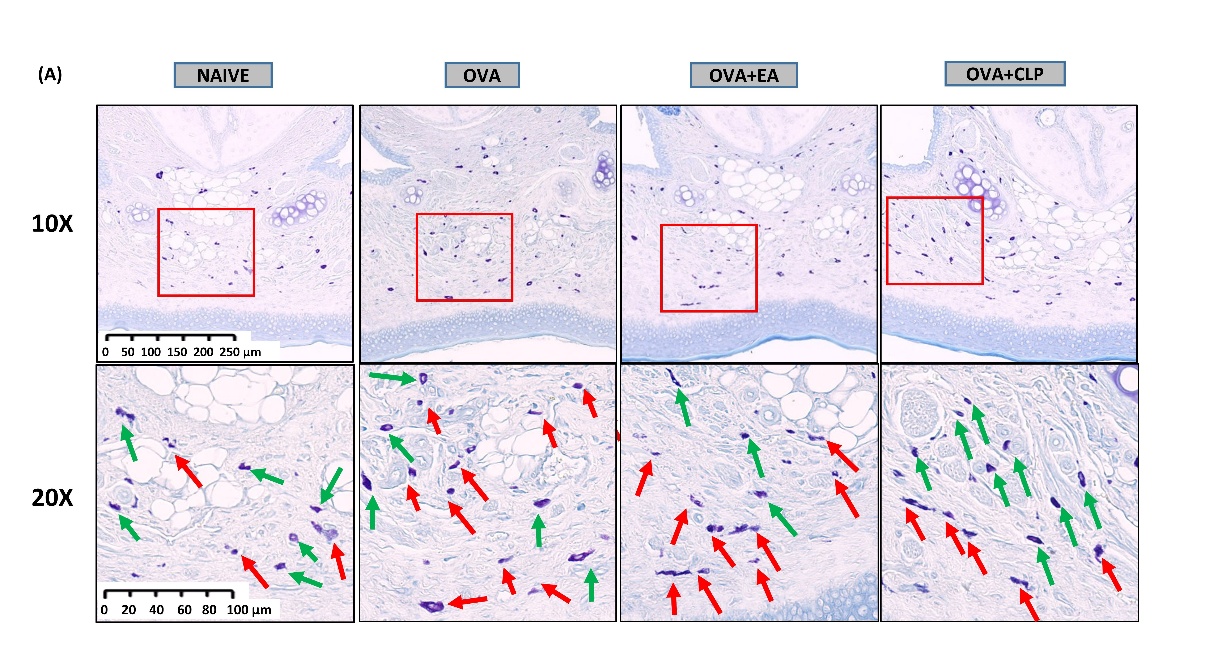


Representative histological images and quantification of mast cell number and degranulation in nasal mucosal tissue following 7-day OVA challenge. OVA increased mast cell number and degranulation compared with naive controls. Chlorpheniramine (CLP) significantly reduced mast cell activation, whereas electroacupuncture (EA) showed a more limited effect (n = 2 mice/group).
